# Inhibition of ERO1a and IDO1 improves dendritic cell infiltration into pancreatic ductal adenocarcinoma

**DOI:** 10.3389/fimmu.2023.1264012

**Published:** 2023-12-22

**Authors:** Apple Hui Min Tay, Riccardo Cinotti, Newman Sui Kwan Sze, Andreas Lundqvist

**Affiliations:** ^1^ School of Biological Science, Nanyang Technological University, Singapore, Singapore; ^2^ Department of Oncology-Pathology, Karolinska Institutet, Stockholm, Sweden; ^3^ Department of Health Sciences, Faculty of Applied Health Sciences, Brock University, St. Catharines, ON, Canada

**Keywords:** ERO1a, IDO1, dendritic cell, PDAC, secretome, spheroid, myeloid cell compartment

## Abstract

**Introduction:**

Pancreatic ductal adenocarcinoma (PDAC) is one of the most lethal and treatment resistant cancers. Due to its desmoplastic and hypoxic nature along with an abundance of myeloid cell infiltration and scarce T cell infiltration, PDAC is considered a cold tumor.

**Methods:**

Here we sought to investigate myeloid cell infiltration and composition in PDAC spheroids by targeting the hypoxia-associated pathways endoplasmic reticulum oxidoreductase 1 alpha (ERO1a) and indoleamine 2,3-dioxygenase 1 (IDO1). Using MiaPaCa2 spheroids with hypoxic core, we assessed the roles of ERO1a and IDO1 inhibition in modulating monocyte infiltration and differentiation, followed by characterizing immunomodulatory factors secreted using LC-MS/MS.

**Results:**

Inhibition of ERO1a and IDO1 significantly improved monocyte infiltration and differentiation into dendritic cells. LC-MS/MS analysis of the PDAC spheroid secretome identified downregulation of hypoxia and PDAC pathways, and upregulation of antigen presentation pathways upon inhibition of ERO1a and IDO1. Furthermore, immunomodulatory factors involved in immune infiltration and migration including interleukin-8, lymphocyte cytosolic protein 1, and transgelin-2, were upregulated upon inhibition of ERO1a and IDO1.

**Discussion:**

Collectively, our results show that inhibition of ERO1a and IDO1 modulates the tumor microenvironment associated with improved monocyte infiltration and differentiation into dendritic cells to potentially influence therapeutic responses in patients with PDAC.

## 1 Introduction

Pancreatic cancer is a highly lethal cancer with limited clinical success of conventional chemotherapy and immunotherapy (1). It is among the most hypoxic tumors with a significant depletion of oxygen compared to its physiological state (2, 3), making it difficult for immune cells to infiltrate the tumor microenvironment (TME). Pancreatic ductal adenocarcinoma (PDAC), the most prevalent subtype of pancreatic cancer, has a multi-faceted TME comprising of large amounts of myofibroblast-like cells and myeloid cells (4). This results in desmoplasia within the hypoxic solid tumor core, creating a barrier to cancer immunotherapy. Moreover, the tumor mutation burden of PDAC is relatively low (5, 6), accompanied with limited infiltration of T effector cells (Teff) (6), leading to an immunosuppressive cold tumor phenotype.

The myeloid cell compartment plays a crucial role in creating the immunosuppressive cold TME in PDAC. Myeloid-derived suppressor cells (MDSCs) and tumor associated macrophages (TAMs) are the predominant immunosuppressive phenotypes that accumulate in PDAC tumors (7–9). MDSCs can suppress Teff and stimulate regulatory T cells (Treg) differentiation (10), while TAMs can inhibit anti-tumor effector functions by expression of co-inhibitory receptors ligand like programmed death ligand 1 (PD-L1) (11). Moreover, cytokines like granulocyte-macrophage colony stimulating factor (GM-CSF) and IL-6 inhibit the maturation of dendritic cells (DCs) (12) and decrease co-stimulatory molecules like CD40 which further promote the immunosuppressive TME in PDAC. Therefore, modulating the myeloid cell compartment presents a favorable approach to enhance the efficacy of immunotherapeutic strategies against PDAC.

Endoplasmic reticulum oxidoreductase 1 alpha (ERO1a), a glycoprotein that mediates oxidative formation of protein disulphide bonds (13), has been shown to implicate immune escape through the regulation of MHC class I expression (14) and the induction of MDSCs (15). ERO1a overexpression has also been associated with poor prognosis in PDAC (16, 17). We previously identified ERO1a to be hypoxia-inducible and involved in tumor formation, where genetic deletion of ERO1a in PDAC tumors reduced PD-L1 expression and prevented tumor formation *in vivo* (17). Our proteomic analysis also revealed an increased tryptophan to kynurenine conversion under hypoxia, indicating an increase in indoleamine 2,3-dioxygenase (IDO) activity. This tryptophan catabolism acts as an immunoregulatory control point (18), where IDO1 is interferon (IFN) inducible that drives immune suppression. It is also harnessed by PDAC through the induction of MDSCs and M2 anti-inflammatory macrophages (19). Hence, targeting ERO1a and IDO1 in PDAC may represent a promising approach to overcome the immunosuppressive TME.

In addition to the cellular interactions within the TME, tumor cells release signaling molecules, collectively known as secretome, into the surrounding extracellular space and influence the TME in an paracrine or autocrine manner (20). In PDAC, the tumor secretome is influenced by its hypoxic nature and cold immunosuppressive phenotype (21, 22). This enhanced secretome plays a crucial role in promoting cancer progression and facilitating immune escape (20, 23, 24). In this study, we sought to investigate the impact of inhibiting the two hypoxia-driven immune checkpoints - ERO1a and IDO1, on the modulation of the myeloid cell compartment in PDAC, using a quantitative proteomic approach and immunophenotyping analysis on three-dimensional spheroid model.

## 2 Materials and methods

### 2.1 Cell culture and treatment

Human pancreatic cancer cell line - MiaPaCa2 (American Type Culture Collection, ATCC) and human myeloid leukemia, pro-monocytic cell line - U937 (ATCC) were maintained in DMEM high glucose complete media supplemented with 10% heat inactivated fetal bovine serum (HI FBS) and 1% penicillin-streptomycin (PS) at 37°C and 5% CO_2_. Trypsin 0.5% with EDTA was added to detach adherent cells after which trypan blue 0.4% (Sigma) viability exclusion staining was performed. Reagents were purchased from Thermo Fisher Scientific unless otherwise stated. ERO1a inhibitor (ERO1ai) - EN460 (ACME Research and Sigma) and IDO1 inhibitor (IDO1i) – Epacadostat (InvivoChem and MedChemExpress) were used at half-maximal inhibitory concentration (IC50) of 1.9uM (25) and 10nM (26) respectively.

### 2.2 PBMC and myeloid cell isolation

Peripheral blood mononuclear cells (PBMCs) were isolated from healthy anonymized blood donors’ buffy coat (Karolinska University Hospital) by Ficoll density gradient centrifugation (Cytiva). CD11b+ myeloid cells were isolated using MACS CD11b MicroBead (Miltenyi Biotec) according to manufacturer’s instruction.

### 2.3 Spheroid formation and immune cell infiltration

Spheroids were formed with MiaPaCa2 cells seeded at 5x10^3^ or 1 x10^4^ cells/well in 96-well ultra-low attachment plate (Corning or Nunclon™) in DMEM-F12 serum-free supplement media containing 1% B27, 10ng/mL basic fibroblast growth factor (bFGF) and 20ng/mL epidermal growth factor (EGF) or 10% HI FBS 1%PS for five days. Carboxyfluorescein succinimidyl ester (CFSE) labelled U937 and CD11b+ myeloid cells at 5x10^4^ and 3x10^4^ cells/well respectively, were added to the spheroid on day five. After three days of co–culture, the spheroid was collected and split into two groups – IN and OUT. IN indicates immune cells infiltrated into the spheroid, while OUT indicates immune cells that did not infiltrated into the spheroids. Spheroids were washed with 2% FBS in PBS (Flow Cytometry, FC buffer) twice before flow cytometry analysis. ERO1ai and IDO1i were added either on day zero (D0) of tumor spheroid formation or day five (D5) simultaneously with myeloid cells.

### 2.4 Flow cytometry analysis

All antibodies used for FC are listed in **Supplementary Table 1**. After spheroid IN and OUT, single tumor and immune cells were washed with FC buffer, cell surface antibodies and live/dead (L/D) marker were incubated with samples at 4°C for 20 minutes in dark. Intracellular staining was performed using eBioscience™ or BD Biosciences fixation and permeabilization set. Samples were washed and resuspended with FC buffer before acquiring on BD LSRFortessa X-20 or NovoCyte Quanteon (ACEA Bioscience). FlowJo software (Tree Star) was used for analysis by gating single cell based on forward and side scatters (**Supplementary Figure 1**).

### 2.5 Secretome collection and processing

**Figure 1:**
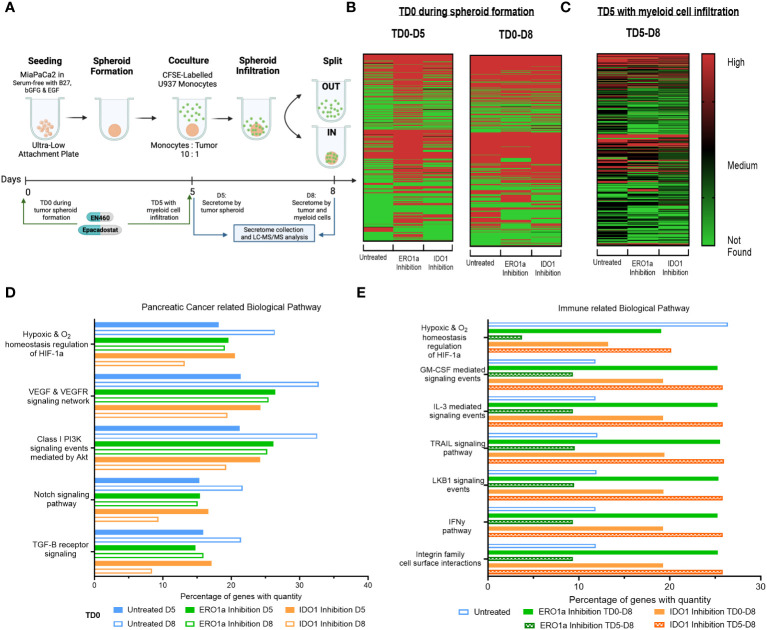
Secretome heatmap and GO analysis of ERO1a and IDO1 inhibition. **(A)** Experimental setup for MiaPaCa2 spheroid formation and U937 myeloid cell infiltration. Heatmap of proteins identified in secretome after treatment at **(B)** day 0 (TD0 during tumor spheroid formation) and collected at day 5 (D5 after tumor spheroid formation) or day 8 (D8 after myeloid cell infiltration), as well as **(C)** inhibition of ERO1a or IDO1 on day 5 (TD5 with myeloid cell infiltration). FunRich quantitative gene ontology analysis in **(D)** pancreatic cancer related biological pathway for TD0 between D5 and D8, and **(E)** immune related biological pathway between TD0 and TD5. All proteins used in all proteomic analysis were identified with FDR ≤0.01 confidence and was a Master Protein present in at least two of three technical replicates. TD0: Treatment on day 0. TD5: Treatment on day 5. D5: Secretome collected on day 5. D8: Secretome collected on day 6.

Conditioned medium of MiaPaCa2 spheroids and immune cell infiltration were collected for secretome analysis on day five (D5) after tumor spheroid formation and on day eight (D8), three days after U937 cell infiltration (**Figure 1A**). Protease and phosphatase inhibitor cocktail (Sigma) were added after collection. Secretome was pooled from 64 and 48 spheroids for treatment at Day zero (D0) and five (D5) respectively. Secretome processing protocol was adapted from established reviews (27) and previously published in-house lab protocols (28, 29). Briefly, secretomes were processed at 4°C with centrifugation of 300 RCF for 10 minutes to remove cells, followed by 2,000 RCF for 10 minutes to remove dead cells. Supernatants were then passed through 0.2uM flitter (Sartorius) to remove additional cells and cell debris. Finally, supernatants were concentrated using ultrafiltration at Amicon Ultra 3kDa Molecular Weight Cut-Off (MWCO; Millipore Merck) following manufacturer’s instructions. Supernatant was collected for 10% SDS-PAGE gel fractionation before in-gel digestion for LC-MS/MS analysis.

### 2.6 Proteomic sample preparation

For in-gel digestion and desalting, 10% SDS-PAGE gel fractionation was first performed by cutting each lane into 5 fractions with fraction 1 (F1) being the smallest MW and F5 the largest MW. All gels were subsequently cut into ~1mm^2^ size, before proceeding to in-gel digestion as previously published (28, 29). Trypsin (Sigma) at 1:100 was added at 0 and 2 hours and incubated at 37°C overnight for at least 16 hours. Peptides were extracted from the digested gel pieces with 50% Acetone Nitrile (ACN) 2% Acetic Acid (AA) in Ammonium Bicarbonate Buffer (ABB) during vortexing for 30 minutes. Extraction step was repeated until the gel pieces turned white. Extracted peptide solutions were centrifuged at 15,000 RCF for 3 minutes before using a gel loading tip to transfer the supernatant to a new Eppendorf tube for SpeedVac dry. Dried digested peptides were reconstituted in 0.1% trifluoroacetic acid (TFA) and vortexed for 30 minutes followed by desalting with Sep-Pak Vac 1cc 50mg C18 Solid Phase Extraction (SPE) cartridge (Waters) was used in accordance with manufacturer’s guidance. Desalted peptides were dried with SpeedVac and stored in -20°C prior to LC-MS/MS analysis.

### 2.7 LC-MS/MS analysis

Desalted peptides were reconstituted in 0.1% formic acid (FA) in 3% ACN for LC-MS/MS analysis in an UltiMate™ 3000 RSLCnano system coupled to a Q Exactive™ Hybrid Quadrupole-Orbitrap™ mass spectrometer as previously described (17, 28–30). Each sample was injected in triplicate into EASY-Spray™ column (75um x 10cm ID Acclaim™ PepMap™ RSLC C18, 3um, 100 A°).

### 2.8 Proteomic bioinformatic analysis

Raw output files from LC-MS/MS were processed using Proteome Discoverer™ software version 2.1.1.21 (PD2.1; Thermo Fisher Scientific) as previously described (30). Protein identification was done by mapping against the UniProt KnowledgeBase (UniProtKB) Homo sapiens protein database (downloaded on 6 Feb 2017, 1,586,247 sequences and 61,972,042 residues) and using SEQUEST-HT and Mascot search engines. For target false discovery rate (FDR), a semi-supervised machine learning – Percolator was used with q-value <0.01 strict and <0.05 relaxed validation on identified peptides. Quantification of identified protein within the sample uses exponentially modified protein abundance index (emPAI) and spectral area. A protein list generated from PD2.1 was sorted to include only Master proteins with FDR < 1% (Exp. q value <0.01). Functional Enrichment Analysis Tool (FunRich) version 3.1.3 (31) was used for quantitative gene enrichment analysis in biological pathway, transcription factor and site of expression. The mass spectrometry proteomics data have been deposited to the ProteomeXchange Consortium via the PRIDE (32) partner repository with the dataset identifier PXD044177.

### 2.9 Secretory interleukin-8 assay

Cell culture supernatants of the above described MiaPaCa2 spheroid and myeloid cells co-cultures were collected on day 8. Sandwich enzyme-linked immunosorbent assay (ELISA) for interleukin-8 (IL-8) was performed according to manufacturer’s instruction (Thermo Fisher Scientific).

### 2.10 Public database analysis

TIMER 2.0 (33) was used to compare differential gene expression between tumor and adjacent normal tissues and predict its correlation with tumor-infiltrating immune cells across The Cancer Genome Atlas (TCGA) - pancreatic ductal adenocarcinoma (PAAD) cohort. Purity adjusted spearman’s rho (Partial Spearman’s Correlation) was used with purity adjustment. Data was exported and plotted with ChitPlot (https://www.chiplot.online/).

### 2.11 Statistical analysis

Experimental replicates are presented as mean ± standard deviation (SD) in bar graph or minimum, first quartile, median, third quartile and maximum in box plot, or as stated in the figure legend of the result section. Grouped statistical analysis – one-way analysis of variance (ANOVA) with multiple comparisons, was performed using Prism 9 (GraphPad Software). The types of statistical analysis are stated in figure legends. A p-value <0.05 was set to consider a difference to be statistically significant with * p<0.05, ** p<0.01, *** p<0.001 and **** p<0.0001.

## 3 Results

### 3.1 ERO1a and IDO1 inhibition downregulate secretome associated with myeloid cell infiltration

To investigate alterations in the secretome by PDAC tumor spheroids upon inhibition of ERO1 or IDO1 at day 0 (TD0), supernatants from treated and untreated tumor spheroids were collected on day 5 (D5) of tumor culture and on day 8 (D8) following three days co-culture with U937 myeloid cells (**Figures 1A, B**, **S2A**). Comparison of D5 and D8 secretomes revealed an upregulation of 149 proteins in D8 compared with D5 secretomes in the untreated condition, indicating that myeloid cells induce changes in the secretome upon infiltration into the tumor spheroid. Additionally, IDO1 inhibition upregulated 81 proteins in the D8 secretome compared to D5, while ERO1a inhibition downregulated 60 proteins upon myeloid cell exposure. Inhibition of ERO1a and IDO1 also resulted in identification of 203 and 65 more secreted proteins at D5 respectively, signifying their inhibition upregulated the tumor spheroid secretome. However, these changes were not observed when comparing D8 secretomes, suggesting that ERO1a and IDO1 primarily affect tumor spheroid rather than the myeloid cells. To further investigate the effect of ERO1a and IDO1 inhibition in the presence of myeloid cells, inhibitors were added on day 5 in the presence of myeloid cells (TD5), and secretomes collected on day 8 were compared (TD5 D8, **Figure 1C**). Overall, ERO1a and IDO1 inhibition led to a reduced number of proteins in the secretome, with 105 and 16 proteins being downregulated respectively (**Supplementary Figure S2B**).

With ERO1a and IDO1 inhibition modulating the secretome to different extents, the proteins identified, and their corresponding abundances were examined using quantitative gene ontology (qGO). Biological pathway qGO analysis revealed 5.54-11.42% upregulation of quantified proteins in hypoxia and pancreatic cancer (PC)-related pathways at D8 compared with D5 in untreated condition (**Figure 1D**). This suggests that the presence myeloid cells render the pancreatic tumor spheroid environment more hypoxic and conditioned to upregulate proteins involved in PC progression. Inhibition of ERO1a or IDO1 prevented this upregulation between D5 and D8, indicating their roles in modulating myeloid cells in PC progression, Although IDO1 inhibition D5 did not downregulate proteins in these PC pathways compared to untreated D5, it downregulated 4.83-8.7% quantified proteins involved in these pathways upon myeloid cell infiltration D8. To further dissect the effects of ERO1a and IDO1 on myeloid cell infiltration into the tumor spheroid, the secretomes at D8 after TD0 (during tumor spheroid formation) and TD5 (with myeloid cell infiltration) using immune-related biological pathways qGO were compared (**Figure 1E**). Inhibition of ERO1a on day 5 reduced hypoxia signaling from 26.4% in untreated spheroids to 3.7%. Myeloid and pro-inflammatory signaling pathways were upregulated 2-fold with ERO1a inhibition at TD0 and IDO1 inhibition at TD5. Collectively, these results highlight the suppressive nature of myeloid cells in the PDAC through upregulating secretome involved in hypoxia and PC progression pathway, where ERO1a and IDO1 inhibition can prevent these changes. Their respective roles in myeloid cell infiltration were demonstrated with ERO1a modulating the hypoxic tumor compartment and IDO1 modulating the myeloid cell compartment.

### 3.2 Combined ERO1a and IDO1 inhibition upregulates antigen presentation biological pathways and transcription factors associated with myeloid cell activity

**Figure 2:**
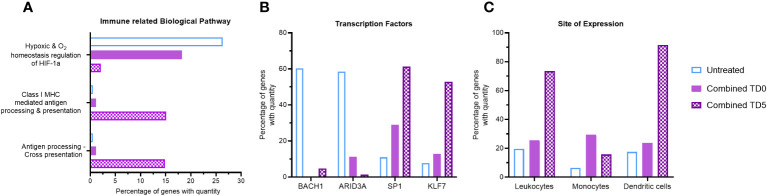
Gene ontology analysis of combined ERO1a and IDO1 inhibition. Combination of ERO1a and IDO1 inhibition quantitative gene ontology analysis in **(A)** immune related biological pathway, **(B)** transcription factors and **(C)** site of expression between TD0 and TD5. All proteins used in all proteomic analysis were identified with FDR ≤0.01 confidence and was a Master Protein present in at least two of three technical replicates.

Given the distinct influence of ERO1a inhibition on the hypoxic tumor secretome and the greater impact of IDO1 inhibition on myeloid signaling, the combined effect of ERO1a and IDO1 inhibition at the same single treatment concentration was investigated for their immune-related biological pathways. No difference in spheroid size was observed between the single treatments and the combined treatment (data not shown). Compared with untreated spheroids, combined inhibition of ERO1a and IDO1 at day 5 resulted in a 12.5-fold downregulation of the hypoxic pathway (2.11% vs 26.38%), while antigen presentation biological pathways were increased by 14.59% (**Figures 2A**, **S3A**). Furthermore, additional immune-related biological pathways including IL-3 and GM-CSF mediated signaling events were also increased upon combined inhibition. Notably, inhibition of either ERO1a or IDO1 alone at day 5 did not influence antigen presentation biological pathways (**Supplementary Figure S3B**). Furthermore, analysis of the transcription factor using qGO revealed the downregulation of BACH1 and ARID3A, which are associated with non-inflammatory M2 macrophages and reduced myeloid lineage (34) respectively, by ERO1a and IDO1 inhibition (**Supplementary Figure S3C**). The combination of ERO1a and IDO1 inhibition at day 5 resulted in a greater reduction compared to untreated controls (5% vs 60% in BACH1, 1.4% vs 59% in ARID3A, **Figure 2B**). Likewise, transcription factors involved in myeloid activity (35, 36) and macrophage activation (37), such as SP1 (Specificity protein 1) and KLF7 (Kruppel like factor 7), were upregulated by the combination of ERO1a and IDO1 inhibition at day 5. These results emphasize the synergistic effects of combined treatment, with a 61% increase in SP1 and a 53% increase in KLF7 compared with 11% and 8% in untreated control (**Figure 2B**).

To gain further insights into the impact of the secretome on immune cell distribution, site of expression qGO analysis was performed. Generally, treatment on day 5 resulted in increased secretion of proteins expressed in leukocytes compared to treatment on day 0 (**Supplementary Figure S3D**), and combined treatment showed a notable enrichment of DCs (92% vs 18%, **Figure 2C**). Collectively, the combination of ERO1a and IDO1 inhibition modulates the secretome and results in upregulation of antigen presentation pathways and promoting the enrichment of DCs, suggesting a more inflamed TME.

### 3.3 Secreted IL-8, LCP-1 and TAGLN2 are associated with upregulation in DC biological pathways

**Figure 3:**
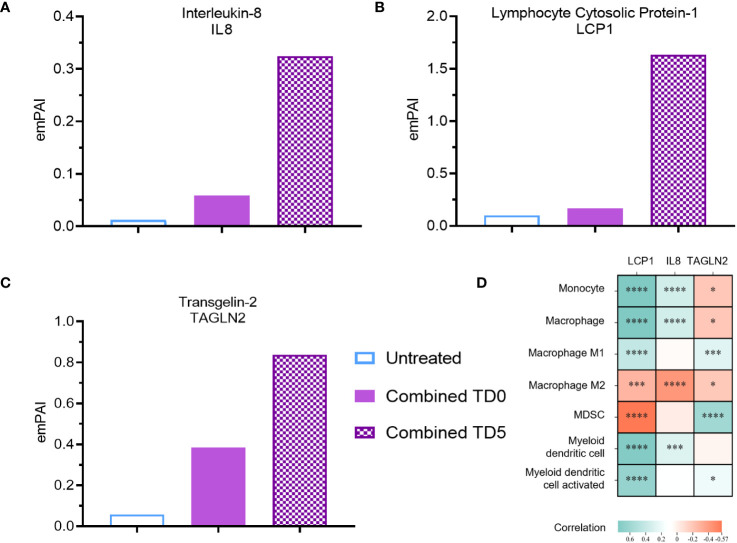
Secreted protein abundance associating with DC infiltration pathways. Secreted protein **(A)** IL8, **(B)** LCP1 and **(C)** TAGLN2 protein abundance (TD0 n = 64, and TD5 n = 48). **(D)** Correlation of corresponding gene expression with myeloid cell infiltration in TCGA-PAAD cohort using TIMER2.0. All proteins used in all proteomic analysis were identified with FDR ≤0.01 confidence and was a Master Protein present in at least two of three technical replicates. emPAI: exponentially modified Protein Abundance Index. Statistically significant spearman’s correlations were indicated with *p < 0.05, ***p < 0.001 and ****p < 0.0001.

To identify putative immunomodulatory factors associated with the increase in DC biological pathways, further analysis using the emPAI was performed. IL-8, a pro-inflammatory chemokine involved in immune infiltration (38), exhibited a substantial 27-fold upregulation with combined inhibition at day 5 (**Figure 3A**). Likewise, inhibition of IDO1 at day 0 resulted in upregulation of IL-8 (**Supplementary Figure S4A**). Lymphocyte cytosolic protein-1 (LCP1) and tansgelin-2 (TAGLN2), both involved in macrophage and DC migration (39–41), were also upregulated by 20-fold and 14-fold respectively upon combined inhibition on day 5 (**Figures 3B, C**). Inhibition of IDO1 but not ERO1a on day 5 also resulted in upregulation of LCP1 and TAGLN2 (**Supplementary Figures S4B, C**). IL-8 was further confirmed with ELISA to be upregulated with combined inhibition at both TD0 and TD5 (**Supplementary Figure S4D**). To validate these findings, the TCGA-PAAD cohort was analyzed and a significant positive correlation between the expression of IL-8 and LCP1 and DC signature was observed (**Figure 3D**).

### 3.4 ERO1a and IDO1 inhibition improves monocytes infiltration and differentiation into dendritic cells

**Figure 4:**
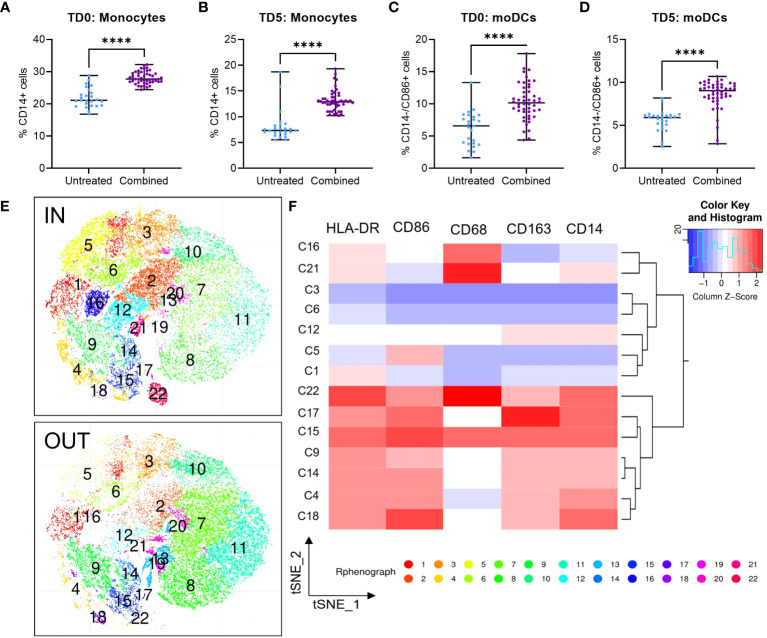
Infiltration of U937 myeloid cells into MiaPaCa2 spheroids. Infiltration of **(A, B)** CD14+ monocytes and **(C, D)** CD14-/CD86+ moDCs U937 cells after treatment at day 0 (TD0, n = 64) and day 5 (TD5, n = 48). **(E)** T-stochastic neighbor embedding (tSNE) analysis within (IN) and outside (OUT) of the spheroids and **(F)** corresponding heatmap for marker expression among myeloid clusters only. Scatter dot plots with median and range are presented. Statistical analysis – unpaired T tests were performed with **** *p* < 0.0001.

To evaluate the immunomodulatory effect of ERO1a and IDO1 inhibition, flow cytometry was used to examine the infiltration and differentiation of U937 myeloid cells into MiaPaCa2 PDAC spheroids. While the inhibition of ERO1a did not influence the infiltration of myeloid cells, inhibition of IDO1 significantly increased the overall infiltration of cells into the spheroid (**Supplementary Figures S5A, B**). The majority of the infiltrated cells were monocytes where inhibition of IDO1 and/or ERO1a significantly improved their infiltration (**Figures 4A, B**, **S5C, D**). Among the infiltrated macrophages, the majority expressed CD163 anti-inflammatory M2 marker (median 82-88%, **Supplementary Figures S4E, F**). Inhibition of ERO1a or IDO1 at day 0 resulted in higher frequencies of HLA-DR-/CD68- MDSCs (median 8.5-11%) compared to inhibition on day 5 (median 3.6-4.6%, **Supplementary Figures S5G, H**).

In line with the secretome qGO results, combination of ERO1a and IDO1 inhibition significantly enhanced the infiltration and differentiation of monocytes and monocyte-derived DCs (moDCs) by 1.54-fold (**Figures 4C, D**) compared to untreated controls and inhibition of ERO1a or IDO1 alone (**Supplementary Figures S5I, J**). Using t-stochastic neighbor embedding (tSNE) analysis, we identified moDC denoted by CD14-/CD86+ expression (cluster 5, C5), predominantly present within the spheroid (**Figures 4E, F**). These findings describe the role of ERO1a and IDO1 inhibition to increase infiltration of DCs into PDAC tumors, thus providing an opportunity to promote inflammatory responses.

**Figure 5:**
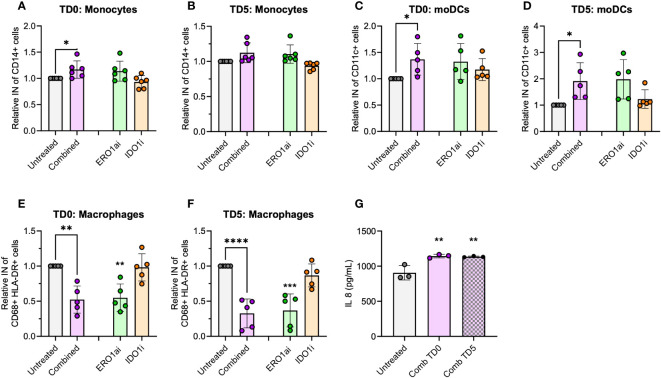
Spheroid infiltration of primary myeloid cells. Relative infiltration of **(A, B)** CD14+ monocytes, **(C, D)** CD11c+ moDCs and **(E, F)** CD68+ HLA-DR+ macrophages after treatment at day 0 (TD0) and day 5 (TD5) (n=5 healthy donors). **(G)** IL-8 secreted in cell supernatant at day 8 (n=3 healthy donors). Bar chart with mean ± standard deviation. Statistical analysis one-way ANOVA with Dunnett’s multiple comparisons were performed with *p < 0.05, **p < 0.01, ***p < 0.001 and ****p < 0.0001.

To enhance the translational relevance of the combined ERO1a and IDO1 inhibition in improving infiltration and differentiation into moDCs, primary PBMCs were used as a source of myeloid cells. Although the overall infiltration of myeloid cells showed no significant differences (**Supplementary Figure S6A**), combined inhibition of ERO1a and IDO1 on day 0 increased total monocytes infiltration by 17% (**Figures 5A, B**). Consistent with the findings in U937 myeloid cells, combined inhibition of ERO1a and IDO1 significantly improved moDC infiltration and differentiation, with treatment on day 0 showing a 36% increase and treatment with ERO1a or IDO1 on day 5 resulted in 1.91-fold increased (**Figures 5C, D**). The majority (mean >80.3%) of the infiltrated cells were DCs with the expression of CD86 (**Supplementary Figure S6B**). With regards to macrophages, the combination and ERO1a inhibition significantly reduced total macrophage infiltration by 47.7-67.4% and 45.4-63.2% respectively (**Figures 5E, F**). This reduction was not observed upon inhibition of IDO1 alone. Although not statistically difference, a trend towards decreased frequency of M1-like macrophages was observed across all treatments (**Supplementary Figures S6C, D**). Combined inhibition at TD0, but not TD5, resulted in a significantly reduced frequency of CD163 positive macrophages, while no statistical significance was observed for double-positive CD206 and CD163 M2-like macrophages (**Supplementary Figures S6E–G**). Immunomodulatory factor, IL-8 identified from the secretome, was also found to be significantly upregulated in both TD0 and TD5 combined inhibition (**Figure 5G**). Altogether, these results show that the combined inhibition of ERO1a and IDO1 increases the infiltration of CD86 positive DC into PDAC spheroids.

## 4 Discussion

PDAC is a highly aggressive and immunosuppressive cancer, posing significant challenges for effective treatments. Its hypoxic and immunosuppressive TME creates barriers for inflammatory responses, where anti-PD1/L1 monotherapy show limited objective response (42). Thus, strategies to overcome such barriers are essential for improving immunotherapy outcomes in PDAC patients. Targeting the myeloid cell compartments, including MDSCs and TAMs, has emerged as a compelling strategy, given their pivotal role in shaping the immunosuppressive TME. In this study, we investigated the role of two hypoxia-driven immune checkpoints – ERO1a and IDO1, in modulating the myeloid cell compartment using a quantitative secretome approach.

Understanding the tumor secretome is essential for exploring immune escape mechanisms, as cancer cells exhibit aberrant secretome profiles influenced by the hypoxic and immunosuppressive TME (20–22, 43). The secretome can induce the expression of the scavenger receptor – MARCO (macrophage receptor with collagenous structure) in MDSCs, suppressing cytotoxic effector functions of Teff and natural killer (NK) cells (44). It also induces an anti-inflammatory status in TAMs through factors like PGE2 (prostaglandin E2) and galectin-9, contributing to T cell exhaustion (30). These secreted factors, together with immunosuppressive MDSCs and TAMs, promote PDAC progression (7–9, 45). Our findings likewise demonstrate the addition of myeloid cells to MiaPaCA2 spheroids upregulate the tumor secretome, activating hypoxia-related pathways and those involved in PC progression, including VEGF (vascular endothelial growth factor), Notch and TGFb (transcription growth factor beta) signaling (46).

ERO1a and IDO1 independently shape the myeloid cell compartment, but their roles in modulating the tumor secretome within myeloid cell interactions are underexplored. In our study, we found that inhibiting ERO1a and IDO1 effectively prevents the upregulation of secretome associated with hypoxia and PC pathways, highlighting their roles as hypoxia-driven immune checkpoints. Additionally, our results corroborate previous findings (17), demonstrating a notable 7-fold reduction in hypoxia signaling in the presence of myeloid cells following ERO1a inhibition.

By targeting both ERO1a and IDO1, we harnessed their unique modulation potential offered on the myeloid cell compartment and the tumor secretome. Previous research has shed light on the distinct roles of ERO1a and IDO1 in influencing the generation of immature myeloid cells, but few studies have explored their modulation of DCs in the TME. ERO1a has been shown to regulate MDSC induction via GM-CSF and CXCL1/2 secretion (15), whereas IDO1 promotes MDSCs and M2 anti-inflammatory macrophages expansion through tryptophan catabolism (19). Particularly, a recent study presented the overexpression of IDO1 inhibited DC maturation and consequently affected immune cell recruitment in an inflammatory liver model (47). Our study identified a 12-fold reduction in hypoxia signaling, emphasizing the potential of combining ERO1a and IDO1 as hypoxia-driven immune checkpoints. Importantly, the combination specifically upregulated antigen presentation pathways, including both MHC class I-mediated and cross-presentation, along with the enrichment of DC site of expression. Our spheroid infiltration model further confirmed improved U937- and healthy PBMC-derived monocytes infiltration and differentiation into moDCs. Considering the role of DCs in facilitating both MHC class I and II antigen presentation, priming both CD4 and CD8 Teff (48), these suggests that combining of ERO1a and IDO1 inhibition promotes an inflamed TME characterized by increased antigen presentation and enhanced DC infiltration. Future studies using immunocompetent *in vivo* models such as the synergistic KPC (Kras^G12D/+^; Trp53^R172H/+^; P48-Cre) mouse model of PDAC can be used to validate these findings.

The 6-fold increase of transcription factors – SP1 and KLF7 gene ontology in response to the combined inhibition of ERO1a and IDO1 underscore their relevance in myeloid cell modulation. It has been reported that SP1 is necessary for myeloid cell activity through CD11b promoter binding (36), while KLF7 promotes macrophage activation via NF-kB (Nuclear factor-kappa B) signaling (37). NF-kB activation in macrophages enhances pro-inflammatory cytokines production and antigen presentation. Therefore, these findings support the potential of combining ERO1a and IDO1 inhibition to promote a pro-inflammatory environment by increasing myeloid cell function and promote pro-inflammatory macrophage polarization.

The characterized upregulation of secreted immunomodulatory factors including, but not limited to IL-8, LCP1 and TAGLN2 provides further support for DC pathway activation in response to the combined inhibition of ERO1a and IDO1. These factors have been implicated in inflammation, immune cell recruitment and activation (38–41). IL-8 was not only detected in the secretome, but also presence in the cell supernatant. It is a pro-inflammatory chemokine that has been shown to attract immune cells to inflammatory sites and promote their activation (38). LCP1, involved in cytoskeleton remodeling, is linked to macrophage migration (39), while TAGLN2 is associated with DC migration and mediated T cell stimulation (40). The upregulation of these factors suggests a favorable immune response characterized by increased DC activation and subsequent immune cells recruitment to the TME. Future studies are needed to complement these insights by employing conventional protein detection methods to strengthen the observed changes in other secreted immunomodulatory factors.

The combined ERO1a and IDO1 inhibition also resulted in the upregulation of several other anti-tumor immune effects. The pro-inflammatory IFNy pathway, crucial for immune cell activation and tumor suppression (49), was upregulated 2.14-fold upon combined inhibition. Additionally, there was an upregulation of tumor suppressor LKB1 signaling events associated with inflammatory cytokines via the STING pathway (50). Moreover, integrin family cell surface interactions, which are crucial for immune cell trafficking into cancerous tissues (51) were also upregulated upon combined ERO1a and IDO1 inhibition. Collectively, these findings indicate that the combination of ERO1a and IDO1 inhibition not only promotes antigen presentation pathways and DC infiltration but also broader anti-tumor immune effects, creating an inflamed tumor microenvironment conducive to mounting an effective immune response against PDAC.

In conclusion, our study provides evidence supporting the simultaneous targeting of ERO1a and IDO1 as a promising strategy for modulating the myeloid cell compartment and altering the tumor secretome in PDAC. Through leveraging on the unique modulation perspectives offered by each checkpoint, significant reductions in hypoxia signaling and specific upregulation of antigen presentation pathways were achieved, accompanied by enhanced infiltration and differentiation of moDC. The observed regulation of key transcription factors, including SP1 and KLF7, along with the upregulation of secreted immunomodulatory factors such as IL-8, LCP1 and TAGLN2, further support the enhancement of myeloid cell function and the promotion of a pro-inflammatory TME. These findings highlight the potential of combined ERO1a and IDO1 inhibition as a promising strategy for reshaping the immunosuppressive TME in PDAC and overcoming immune barriers. Thereby providing valuable insights for the development of targeted immunotherapies that harness the immune system’s potential to combat PDAC. Further investigation is warranted to elucidate the precise mechanisms underlying their modulation of the tumor secretome and their effects on other immune cell populations, particularly the cytotoxic effector cells. These will deepen our understanding of the immunosuppressive TME and guide the advancement of precision immunotherapy approaches.

## Data availability statement

The datasets presented in this study can be found in online repositories. The names of the repository/repositories and accession number(s) can be found below: Proteomics IDEntifications (PRIDE) with dataset identifier PXD044177.

## Ethics statement

Buffy coat from anonymized adult healthy blood donors was obtained from Karolinska University Hospital Blood Bank. Ethical approval was not required for the studies on humans in accordance with the local legislation and institutional requirements because only commercially available established cell lines were used.

## Author contributions

AT: Conceptualization, Data curation, Formal analysis, Funding acquisition, Investigation, Methodology, Software, Supervision, Validation, Visualization, Writing – original draft, Writing – review & editing. RC: Data curation, Investigation, Writing – review & editing. NS: Methodology, Resources, Writing – review & editing. AL: Conceptualization, Funding acquisition, Methodology, Project administration, Resources, Supervision, Writing – review & editing.
