# Supplementary material for: Inhibition of ERO1a and IDO1 improves dendritic cell infiltration into pancreatic ductal adenocarcinoma
Source: Front Immunol. 2023 Dec 22;14:1264012. doi: 10.3389/fimmu.2023.1264012 (PMC10766682; doi:10.3389/fimmu.2023.1264012)
Supplement: Supplementary file 1 [file DataSheet_1.pdf]

Supplementary Material

Supplementary Table 1. List of antibodies used in flow cytometry. **A.** U937-myeloid cells and **B.** PBMC CD11b isolated myeloid cells.

Antigen	Conjugate	Vendor	Catalog Number	A	B
CD14	APC	Thermo Fisher Scientific	17-0149-42	√	
	Pacific Blue	BioLegend	367122		√
CD68	BV 421	BioLegend	301807	√	
	AF647		333819		√
HLA-DR	PerCP Cy5.5	Thermo Fisher Scientific	45-9956-42	√	
	Brilliant Violet 650	BD Bioscience	564231		√
CD86	PE-Cy7	Thermo Fisher Scientific	25-0869-42	√	
	PE-Cy5		15-0869-42		√
CD163	PE	BioLegend	333606	√	
	APC/Fire™ 750		333634		√
CD206	PE-Cy7	BioLegend	141720		√
CD11c	PerCP Cy5.5	BioLegend	337210		√
CD15	PE	BioLegend	323006		√
Live/Dead marker	Aqua	Thermo Fisher Scientific	L34966	√	√

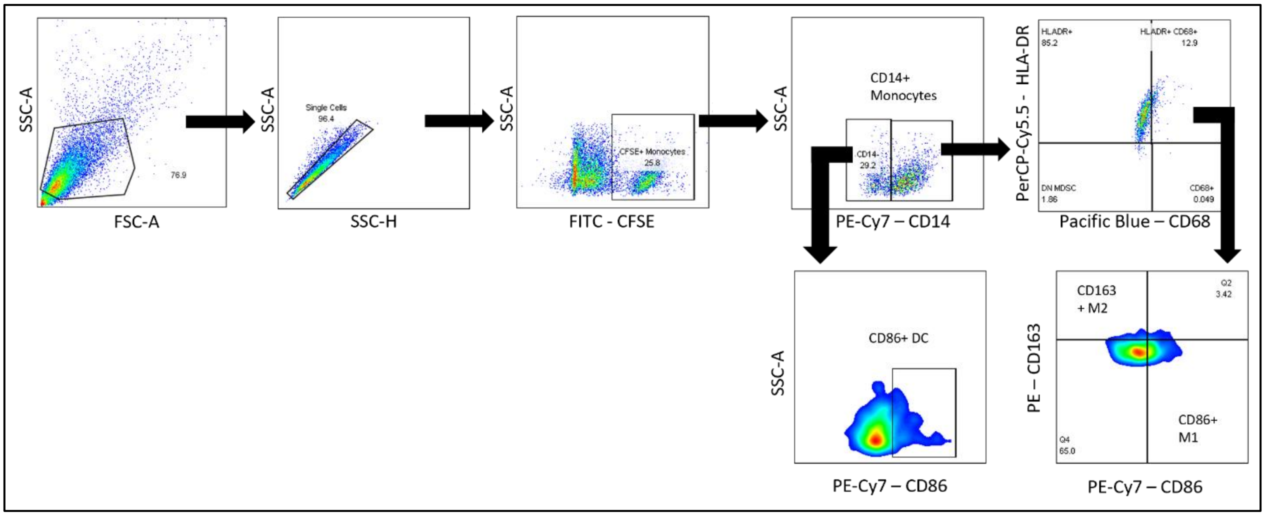

Supplementary Figure 1. Gating strategy for myeloid cell infiltration using single cell based on forward and side scatters.

A.

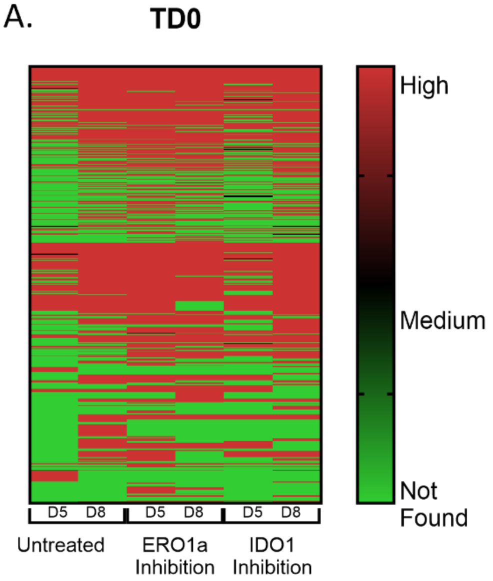

B.

Treatment at Day	Day 0 (TD0)		Day 5 (TD5)
Secretome collected at	Day 5 (D5)	Day 8 (D8)	Day 8 (D8)
Untreated	228	377	583
ERO1a Inhibition	431	371	478
IDO1 Inhibition	293	374	567
Combined	420	412	416

Supplementary Figure 2. (A) Heatmap of proteins identified in TD0 secretome. **(B)** Table summary of secretome protein identified. All proteins used in all proteomic analysis were identified with FDR ≤0.01 confidence and was a Master Protein present in at least two of three technical replicates. TD0: Treatment at day 0, TD5: Treatment at day 5, D5: Secretome collected at day 5, D8: Secretome collect at day 8.

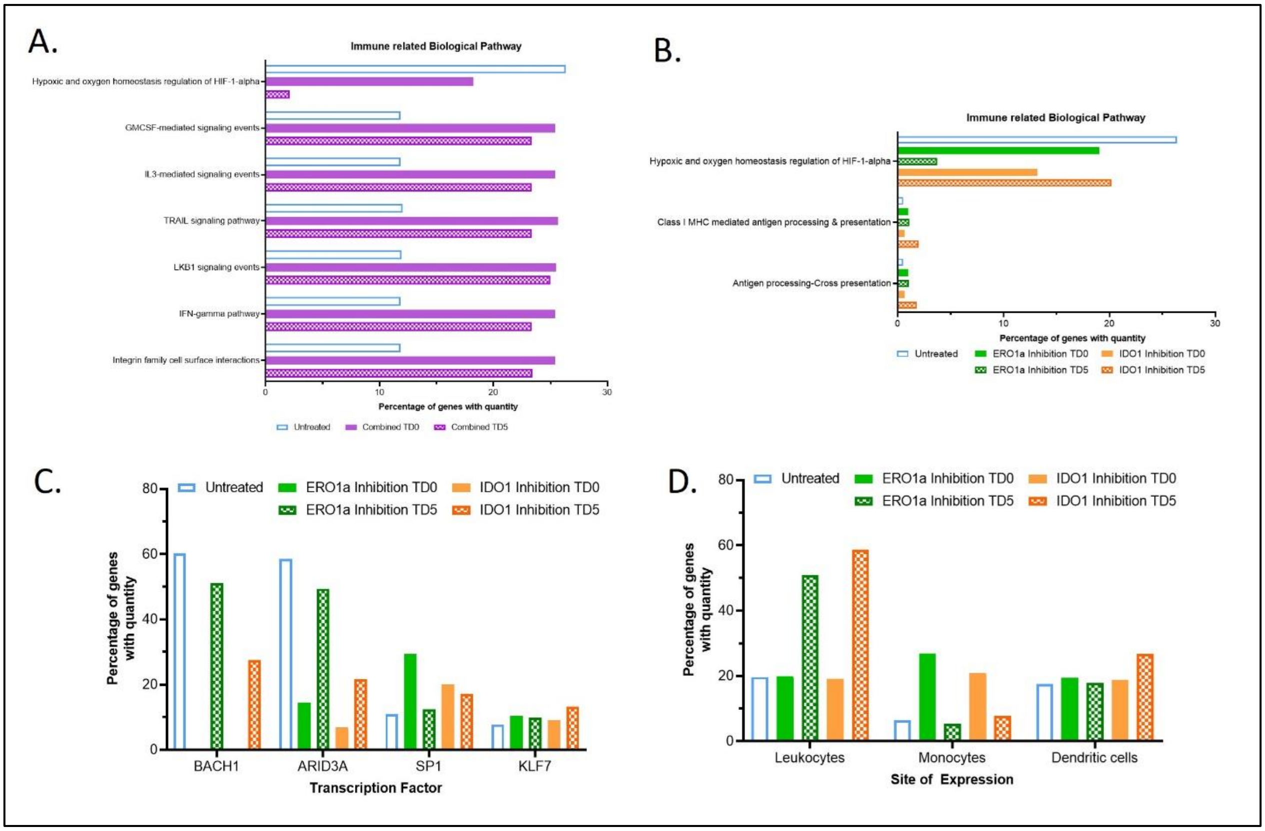

Supplementary Figure 3. FunRich quantitative gene ontology analysis in **(A-B)** immune related biological pathway, **(C)** transcription factor and **(D)** site of expression. All proteins used in all proteomic analysis were identified with FDR ≤0.01 confidence and was a Master Protein present in at least two of three technical replicates.

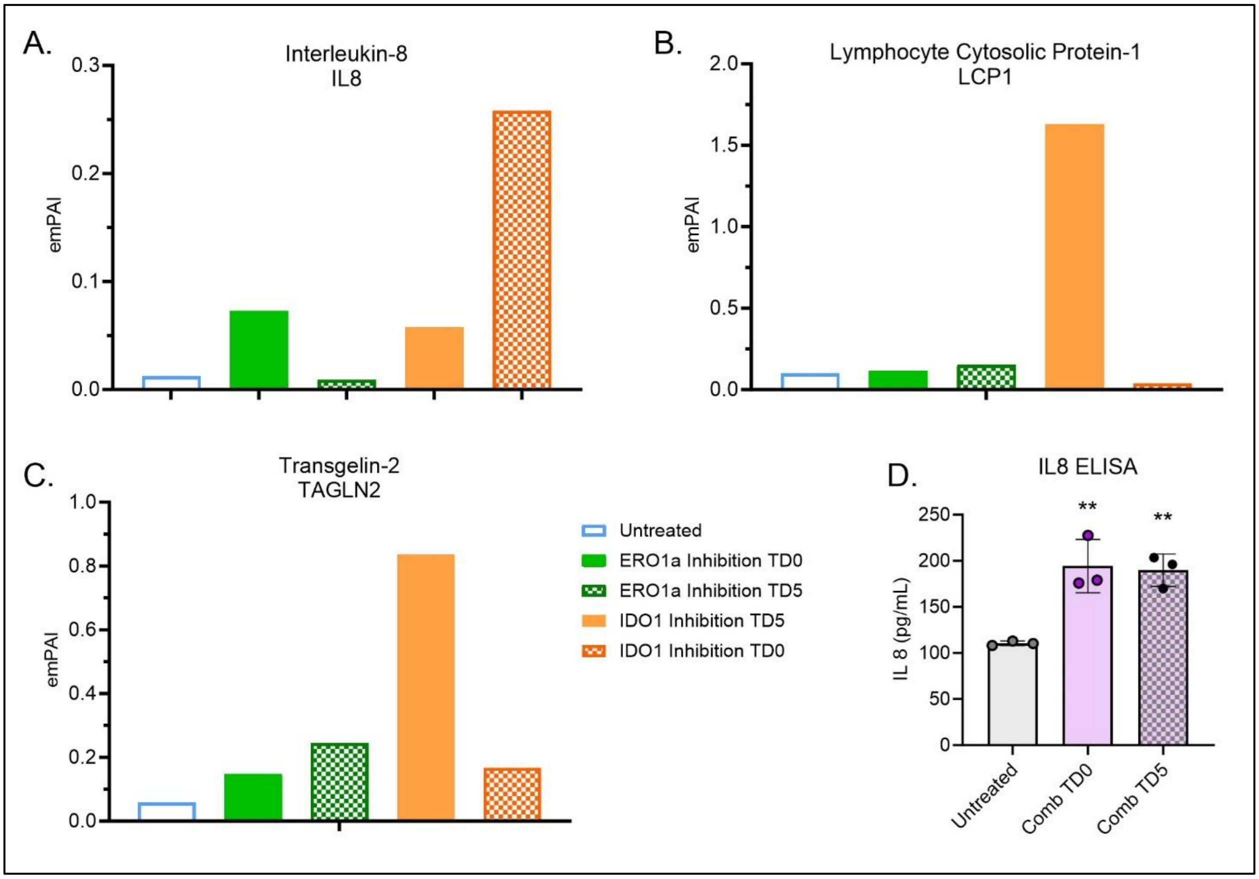

Supplementary Figure 4. Secreted protein abundance of **(A)** IL8, **(B)** LCP1 and **(C)** TAGLN2. **(D)** IL-8 secreted in cell supernatant at day 8 (n =3). All proteins used in all proteomic analysis were identified with FDR ≤0.01 confidence and was a Master Protein present in at least two of three technical replicates. Bar chart with mean ± standard deviation. Statistical analysis one-way ANOVA with Dunnett's multiple comparisons were performed with $**p < 0.01$. emPAI: exponentially modified Protein Abundance Index

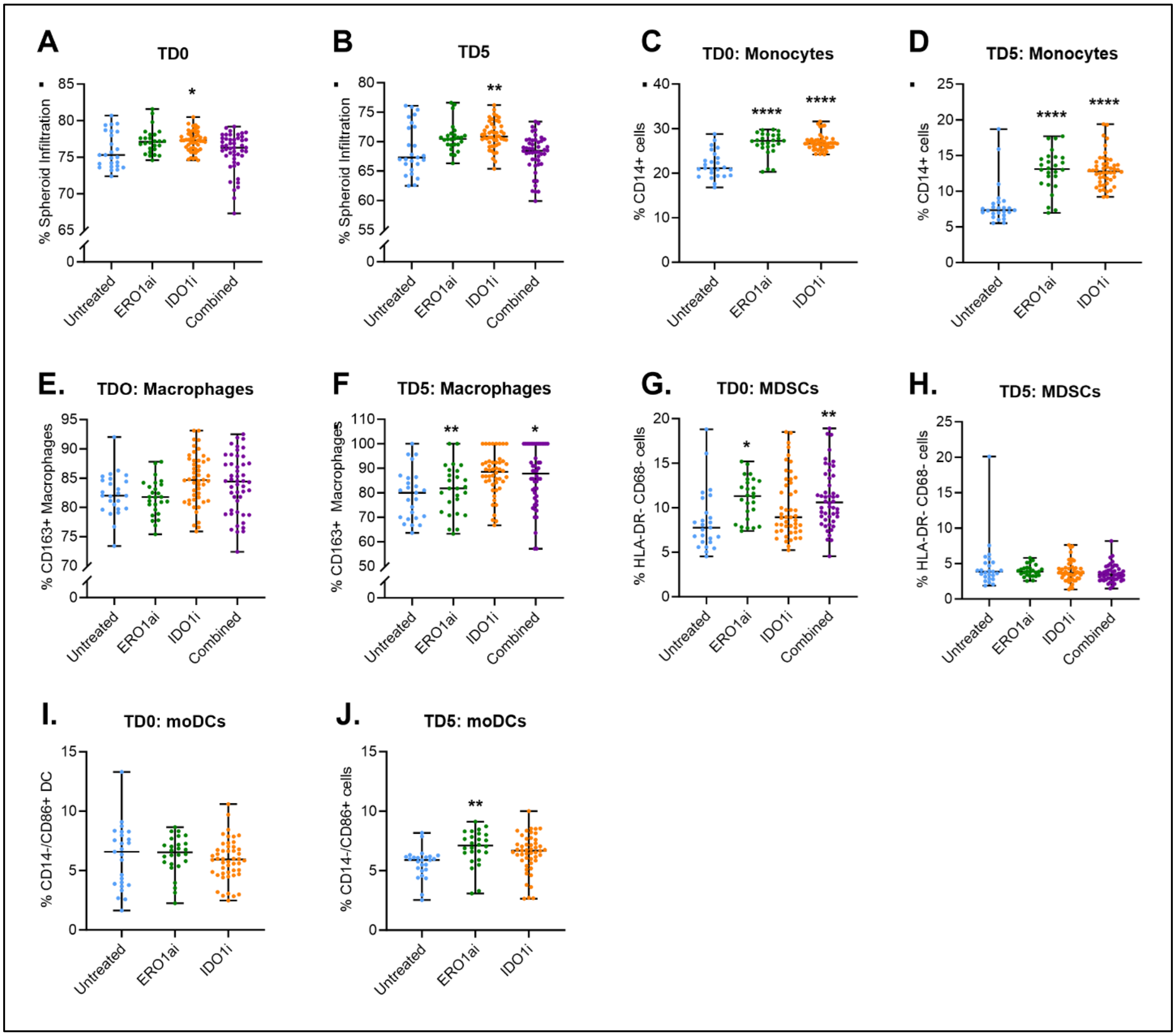

Supplementary Figure 5. Infiltration of (**A-B**) total U937 cells, (**C-D**) CD14+ monocytes, (**E-F**) CD163+ macrophages, (**G-H**) HLADR- CD68- MDSCs, and **(I-J**) CD14-/CD86+ moDCs after treatment at day 0 (TD0, n = 64) and day 5 (TD5, n = 48). Scatter dot plots with median and range are presented. Statistical analysis – one-way ANOVA with Dunnett's multiple comparisons were performed with *$p < 0.05$, **$p < 0.01$, ***$p < 0.001$ and **** $p < 0.0001$.

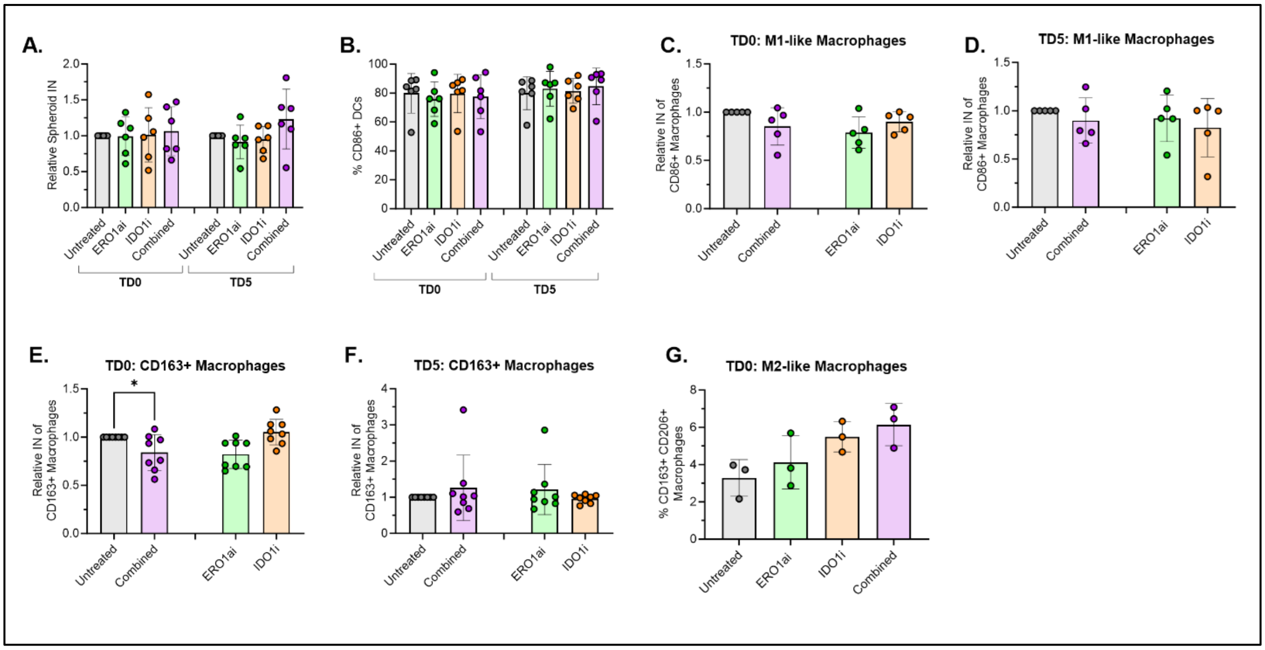

Supplementary Figure 6. Infiltration of (**A**) total myeloid cells (n=5), (**B**) mature moDCs (n=5), (**C-D**) CD86+ M1-like macrophages (n=5), (**E-F**) CD163+ (n=8) and **(G)** CD163+ CD206+ (n=3) M2-like macrophages after treatment at day 0 (TD0) and day 5 (TD5). Bar chart with mean ± standard deviation. Statistical analysis one-way ANOVA Dunnett's multiple comparisons were performed with **p* < 0.05, ***p* < 0.01 and ****p* < 0.001.
